# ChatGPT-4 generates orthopedic discharge documents faster than humans maintaining comparable quality: a pilot study of 6 cases

**DOI:** 10.2340/17453674.2024.40182

**Published:** 2024-03-21

**Authors:** Guillermo Sanchez ROSENBERG, Martin MAGNÉLI, Niklas BARLE, Michael G KONTAKIS, Andreas Marc MÜLLER, Matthias WITTAUER, Max GORDON, Cyrus BRODÉN

**Affiliations:** 1 Department of Orthopedic and Trauma Surgery, University Hospital Basel, Switzerland; 2 Karolinska Institute, Department of Clinical Sciences at Danderyd Hospital, Stockholm; Sweden; 3 Department of Surgical Sciences, Orthopedics, Uppsala University Hospital, Uppsala, Sweden

## Abstract

**Background and purpose:**

Large language models like ChatGPT-4 have emerged. They hold the potential to reduce the administrative burden by generating everyday clinical documents, thus allowing the physician to spend more time with the patient. We aimed to assess both the quality and efficiency of discharge documents generated by ChatGPT-4 in comparison with those produced by physicians.

**Patients and methods:**

To emulate real-world situations, the health records of 6 fictional orthopedic cases were created. Discharge documents for each case were generated by a junior attending orthopedic surgeon and an advanced orthopedic resident. ChatGPT-4 was then prompted to generate the discharge documents using the same health record information. The quality assessment was performed by an expert panel (n = 15) blinded to the source of the documents. As secondary outcome, the time required to generate the documents was compared, logging the duration of the creation of the discharge documents by the physician and by ChatGPT-4.

**Results:**

Overall, both ChatGPT-4 and physician-generated notes were comparable in quality. Notably, ChatGPT-4 generated discharge documents 10 times faster than the traditional method. 4 events of hallucinations were found in the ChatGPT-4-generated content, compared with 6 events in the human/physician produced notes.

**Conclusion:**

ChatGPT-4 creates orthopedic discharge notes faster than physicians, with comparable quality. This shows it has great potential for making these documents more efficient in orthopedic care. ChatGPT-4 has the potential to significantly reduce the administrative burden on healthcare professionals.

Administrative demands in healthcare significantly contribute to surging healthcare expenses, low job satisfaction, and ultimately physician burnout [1]. Evidence suggests that doctors spend an equivalent amount of time on administrative tasks, such as drafting progress notes and discharge documents, requesting labs and analyzing results, as they do in patient interactions [2]. Administrative costs in the United States account for roughly 31% of total healthcare expenditures, translating to $569 billion when adjusted for current medical inflation [3,4].

The recent introduction of large language models (LLMs) such as ChatGPT (OpenAI, 2022) have already had an impact on healthcare in medical education and clinical decision-making [5], medical licensing examinations [6-8], addressing medical inquiries from patients [9-11], and even crafting scientific articles [12,13].

These LLM models could potentially significantly reduce administrative tasks in healthcare by producing patient-specific texts tailored to the physician documentation requirements. The aim of our study is to explore the capabilities of LLM, specifically ChatGPT-4, by evaluating their quality and efficiency in generating discharge documents compared with physicians.

## Methods

### Study setting

6 fictional orthopedic and trauma cases were written by 2 orthopedic surgery residents from two university hospitals from different European countries (3 cases from Sweden and 3 from Switzerland). All health information relevant to the case, including past medical history, radiology reports, laboratory values, progress notes, and interdisciplinary consultation notes were included (see Supplementary data). Although fictional, the cases were thoroughly devised and reviewed by senior orthopedic surgeons to accurately represent a real-life clinical scenario.

The study is reported according to the Consort-AI extension guidelines [14].

### Human-generated discharge documents

A junior attending orthopedic surgeon and a senior orthopedic surgery resident independently created 2 discharge documents for 3 cases each: a discharge summary and a discharge letter, setting the gold standard. The discharge summary is aimed at medical professionals, generally family physicians or general practitioners, who are responsible for the post-discharge care. Using technical medical language, it provides a comprehensive account of the patient’s hospitalization, diagnosis, history of the present illness, procedures undertaken, clinical summary, and the postoperative and discharge guidelines. In contrast, the discharge letter is aimed for the patient; it seeks to translate the information in the discharge summary into layman’s language, so that it can be understood by the patient. In our institutions, clinicians use voice recording for generating discharge summaries. Medical secretaries transcribe these recordings. Upon transcription, clinicians receive the notes for review and approval. In contrast, the discharge letter is written directly by the clinician to ensure the patient obtains the letter without any delays. We diligently logged the duration in the creation of both the discharge summary and letter. Both discharge documents were generated following the standard format of each hospital.

### ChatGPT-4 generated discharge documents

Subsequently, the junior attending orthopedic surgeon and the orthopedic surgery resident who initially generated the discharge documents used a specific prompt for ChatGPT-4 to generate the AI-generated discharge documents. Both physicians were unfamiliar with ChatGPT-4 at the time. This prompt was formulated by 2 authors who are proficient with ChatGPT-4. An additional fictional case, which was excluded from the analysis of the study, was created for this purpose. This prompt was applied uniformly across all cases, with minor modifications to accommodate the distinct formats of Swiss and Swedish discharge notes (see Supplementary data). The time required to generate both the discharge summary and the letter using ChatGPT-4 was documented. The version of ChatGPT-4 used in this study was last updated in September 2023.

### Evaluation of discharge documents

Both human and GPT-4-generated documents were assessed by an expert panel of orthopedic residents and surgeons, blinded to the author of the document. The expert panel comprised 15 orthopedic surgeons (3 senior residents, 6 consultants, and 6 senior consultants). 6 orthopedic surgeons were from Switzerland (2 consultants and 4 senior consultants) and 9 from Sweden (3 residents, 4 consultants, 2 senior consultants). Swiss surgeons assessed cases from Switzerland, whereas Swedish surgeons evaluated cases from Sweden. The panelists independently evaluated the documents using specifically devised quality assessment criteria focusing on the accuracy of the medical information (diagnosis, history of present illness, hospital course, and discharge plan), the suitability of the language for the intended reader (medical professional or patient), conciseness of the text, the presence of factually incorrect, inconsistent, or nonsensical text (hallucinations), and the suitability for clinical use. Additionally, each panelist awarded a subjective quality score from 1–100 to each document (see Supplementary data). The Quality Assessment criteria were developed by our group by adapting an already established framework previously published by Singhal et al. for assessing medical LLM responses to open-ended questions [15]. 4 answer options were provided for each question. The responses were then translated into scores on an ordinal scale of 100. A higher score indicates better performance. The time required for human and GPT-4 document generation was then compared.

### Statistics

Due to the small sample size, we employed descriptive statistics and 95% confidence intervals (CI) to evaluate and compare the quality assessment scores and the time required to generate the human-generated against the GPT-4-generated discharge documents.

### Ethics, registration, data sharing plan, funding, and disclosures

Considering the study’s exploratory nature and the use of fictitious data without real patient information, ethics committee approval was not sought. Additionally, this research project did not receive any financial support. The authors do not have any conflicts of interest to declare. Complete disclosure of interest forms according to ICMJE are available on the article page, doi: 10.2340/17453674.2024.40182

## Results

**Figure 1:**
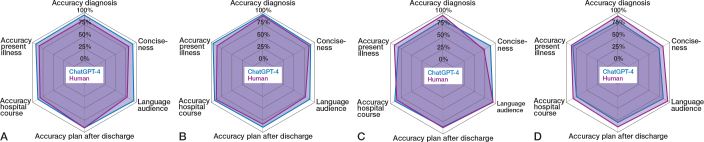
Quality assessment of discharge summaries and letters: A. Swedish summary; B. Swedish letter; C. Swiss summary; D. Swiss letter.

**Figure 2:**
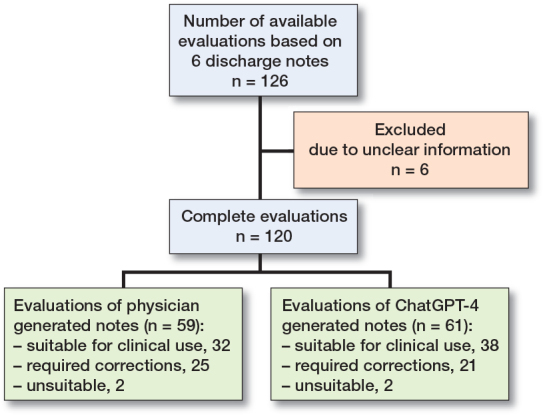
Flowchart of evaluations of discharge notes by the expert panel.

Our study revealed that, overall, discharge documents generated by ChatGPT-4 are of similar quality to those written by physicians. For the Swedish discharge documents, ChatGPT-4 showed a minor edge in quality compared with the physician-generated versions. However, for the Swiss letters, the physician-crafted versions displayed a minor advantage in quality over those generated by ChatGPT-4 (Figure 1). The expert panel conducted a total of 126 evaluations on these discharge documents. 6 of the evaluations could not be used due to unclear information or incomplete information in the evaluation form (Figure 2). 59 evaluations were performed on the physician-generated notes and 61 evaluations on ChatGPT-4 generated notes. Of the documents produced by ChatGPT-4, most (38 assessments) were deemed suitable for clinical use. Some required corrections (21 assessments), while a small number (2 assessments) were deemed unsuitable for clinical use. In contrast, human-generated notes were evaluated 32 times as suitable for clinical use, received 25 indications for corrections, and were considered unsuitable for clinical use on 2 occasions.

Of the 120 evaluations, the panel identified 4 instances of hallucinations in ChatGPT-4-crafted content, in contrast to 6 in human-generated documents.

Regarding subjective quality scores, ChatGPT-4 generated discharge summaries were awarded an average score of 80.7 (CI 76.2–85.2) while those human-generated scored an average 74.3 (CI 68.0–80.7). For discharge letters, ChatGPT-4-generated documents averaged 77.8 (CI 71.1–84.6), while human-generated letters scored 77 (CI 69.3–84.8).

The mean differences in subjective scores between ChatGPT 4 and the physician-produced discharge notes was 6.5 (CI –8.9 to 21.9), and the difference for the discharge letters was 2.9 (CI–14.8 to 20.6)

Discharge documents produced by ChatGPT-4 were generated, on average, 10 times faster than those created by physicians (Table).

**Table:** Time in minutes for physician and ChatGPT-4 to generate discharge notes

Case number	Physician-generated notes	ChatGPT-4-generated notes
Swedish		
Case 1	29.2	3.8
Case 2	33.4	2.9
Case 3	30.7	3.2
Swiss		
Case 1	27.5	3.0
Case 2	22.0	2.4
Case 3	24.0	2.1

## Discussion

This represents the first study to explore ChatGPT-4’s capability in generating discharge documents in the field of orthopedic surgery. We showed that ChatGPT-4 creates orthopedic discharge notes faster than physicians, with comparable quality.

Our findings are in accordance with a recently published study that showed similar results, but focusing on the possibility to generate clinical letters to patients with a high overall correctness and humanness score with ChatGPT [16].

Our findings suggest that the discharge documents produced by ChatGPT-4 overall align closely with the quality of those authored by the orthopedic surgery physicians. A notable difference in quality was observed in Swedish case 2, where ChatGPT-4 appeared to outperform the physician in generating the discharge summary and letter. However, in Swiss case 3, the physician-generated discharge letters obtained higher scores than those produced by ChatGPT-4. Wimsett et al. delineated the fundamental components of effective medical discharge documents, encompassing diagnosis, administered treatments, test results, and discharge guidelines [17]. Nonetheless, several studies indicate that physician-authored documents frequently omit or misrepresent these essential elements [17-18]. The quality of discharge documents is crucial as it influences ongoing patient care and safety and even reimbursement schemes [19].

The subtle variation of human-generated discharge documents performance between the Swiss and Swedish cases might be due to differences in standards and expertise of the physicians creating the discharge notes, but also on average a slightly more senior expert panel evaluating the Swiss cases.

The elevated subjective satisfaction associated with ChatGPT-4-generated compared with the human-generated notes might be attributable not only to the quality of the content but also to its clear and structured linguistic presentation.

Of the documents produced by ChatGPT-4, the majority were deemed suitable for clinical use; some required corrections, while a small number were deemed unsuitable for clinical use. Notably, the ChatGPT-4-generated notes were subjected to evaluation without prior human review in this study. This emphasizes the need for review by a physician after the note is generated by Chat-GPT-4 before use in the medical context.

Within the clinical context of our study, we discerned 4 instances of hallucinations in the ChatGPT-4-generated content, in contrast to 6 such instances in the physician-generated notes. ChatGPT-4 has previously encountered scrutiny due to its potential for generating misleading or fabricated content, termed “hallucinations,” particularly in the context of generating false references in academic articles [20]. Our findings suggest that ChatGPT-4 may, in fact, in the clinical context yield fewer inaccuracies than traditional methods.

ChatGPT-4 generated discharge documents roughly 10 times faster than human writers. This efficiency could free up clinicians to focus on other clinical tasks and may reduce physician burnout [2,21-23]. However, it is important to mention that the review and approval of ChatGPT-4-generated notes may be more time-consuming than assessing notes written by physicians. This can be attributed to clinicians’ active engagement in composition during the note creation process using traditional methods. This dimension was not examined in our current study.

Creating the right prompts for ChatGPT-4 usually requires repeated adjustments. Working closely with researchers familiar with these LLMs can help improve the results. As this area of study grows, we are seeing new specialties emerge, such as “prompt engineering.” Upcoming versions of ChatGPT-4 may come with ready-to-use prompts or models better adjusted to medical terms, which could speed up document creation. Also, if ChatGPT-4 is trained using specific medical data, it might reduce the need for custom prompts, making the process even more efficient [24]. Our study used cases from Sweden and Switzerland to determine whether ChatGPT-4 can adjust to different styles of discharge notes in various countries. However, as these notes were written in English, it remains unclear how well ChatGPT-4 would perform with healthcare documents in other languages.

To fully leverage the benefits of LLMs in clinical settings, we envision the use of a local LLM capable of running in the hospital’s existing IT environment. This approach would possibly ensure that LLMs can effectively and safely interact with the hospital’s electronic health records.

### Limitations

First, we worked with a limited number of fictional orthopedic cases, as this was an initial exploration of ChatGPT-4 capabilities. Second, we deliberately excluded discharge medication from our study due to variations in prescription practices and formats across medical centers. We advocate for a detailed human review of discharge medication. Third, the gold standard was established by the discharge notes generated by 2 orthopedic physicians. Thus, the diverse writing styles, standards, and competences found within a broader group of clinicians were not fully captured. Additionally, it is worth noting that the discharge notes were generated in English, which is not the resident’s native language. However, the evaluation of ChatGPT-4 went beyond the mere examination of written notes by physicians; it also involved a comparison with specific quality criteria. Most of the notes generated by ChatGPT-4 were found to be appropriate and of sufficient quality for use in clinical settings. Another limitation of our study was that evaluators were required to choose from a given Likert scale or answer “yes” or “no” to specific questions. While providing detailed explanations was optional, this approach resulted in a lack of further analysis of types of misinformation and hallucination instances. Nonetheless, the comments received did highlight issues with language use and some misleading phrases in the patient letters generated by GPT-4. Evaluators pointed out a few times that the language in the discharge plans could be confusing. This confusion encompassed areas like unclear directives for initiating anticoagulants, an erroneous distinction between intravenous and oral antibiotics, and 2 instances of vague instructions for referrals to other healthcare services. Consequently, a comprehensive review of AI-generated discharge notes is essential before considering their use in clinical settings. This evaluation could initially be conducted by a physician and, in the long term, potentially by another AI.

Finally, for clinicians already acquainted with ChatGPT-4, potential biases might arise if they were able to distinguish between AI-generated and physician-created notes by the structure and grammar of the note. This recognition could influence our results, as the participants would then not be fully blinded in the study.

### Conclusion

Our pilot study indicates that ChatGPT-4 creates orthopedic discharge papers faster than physicians, with comparable quality, and might assist in reducing the administrative load on orthopedic surgeons by generating clinically suitable discharge documents.

In perspective, while ChatGPT-4 shows promise in producing discharge documents, its use requires careful handling. Entering patient data into the chat might violate Patient Health Information Protection regulations, resulting in substantial fines for unauthorized disclosures [25]. Currently, ChatGPT-4 is not ready for immediate clinical application using patient data because it presents legal and technical challenges that must first be addressed. Further research is needed using patient data from electronic health records. Additionally, exploring the model’s fine-tuning and simplify the prompting process would be beneficial.

### Supplementary data

Cases, evaluation form, prompt, and generated notes are available as supplementary data on the article page, doi: 10.2340/17453674.2024.40182

## Supplementary Material



## Notes

GSR, MM, CB, and MG were responsible for the study conception and designed the study. NB and MW created the cases. MK and MW wrote the discharge notes and prompted ChatGPT-4 to generate the AI documents. GSR and CB created the prompt. GSR and CB wrote the manuscript with continuous feedback from MG, MM, MW, MK, and AMM. MG and CB supervised the study.

The authors would like to express their sincere gratitude to Kenneth Jonsson, Mona Mili, Viktor Schmidt, Sara Lindmark, Fredrik Peyronson, Hannah Coudé Adam, Marc-Antoine Burch, Birgit Oberreiter, Franziska Eckers, Sebastian Müller, Cornelia Baum, and Petros Ismailidis for their assistance in evaluating these cases.

Handling co-editor: Taco Gosens

*Acta* thanks Olivier Groot and Walter van der Weegen for help with peer review of this manuscript.
