# Supplementary material for: ChatGPT-4 generates orthopedic discharge documents faster than humans maintaining comparable quality: a pilot study of 6 cases
Source: Acta Orthop. 2024 Mar 21;95:152–6. doi: 10.2340/17453674.2024.40182 (PMC10959013; doi:10.2340/17453674.2024.40182)
Supplement: Supplementary file 1 [file ActaO-95-40182-s1.pdf]

Supplementary material

A. Cases Switzerland

1. Case 1: Elderly lady with multiple injuries

Hospital for Orthopedic Surgery

Patient Name: Smith, Mary

Gender: female

Age: 77 yrs

Date of Admission 01.01.2023

01.01.2023

Diagnosis:

1. Pertrochanteric femur fracture left
2. Intra-articular distal radius fracture right (dominant)

Admission Note from Emergency Department from 12.02.2024:

History of present illness

A 77 year-old female patient was brought by ambulance from her home after a non-witnessed fall after going to the toilet. The son, who heard her fall, found the patient conscious and alerted the nursing staff. Currently, she cannot say exactly what happened. She complains of a hematoma on the left temple and pain in the right forearm and wrist and left hip.

Past Medical History:

1. Arterial Hypertension

Current medication:

Amlodipin 5mg 0.5-0-0

Torasemid 10mg 0.5-0-0

Candesartan 8mg 1-0-0

Social:

The patient lives on own house with her son and husband. She is independently mobile and has a rollator for longer walking distances outside. Regarding daily activities, the patient does not cook, or go shopping. Food is either bought by the son or gets delivered. The son does the house chores. Patient spends most her time at an elderly day care. She is picked up daily.

Initial Assesment:

SpO2: 97 %; P: 89 /min; BP: 163 /96 mmHG; Temp: 36 °C; AF: 16 /min; O2 GCS 15, Pain VAS 9/10

Right wrist: swelling and tenderness, skin intact. ROM non-evaluable due to pain, hand extension/flexion of all fingers possible with unrestricted strength, neurologically intact.

Left hip: skin intact, no redness, no swelling, no hematoma visible, tenderness over the trochanteric major, extension/flexion of the hip not possible due to pain, leg shortened on the left side, externally rotated, sensory function normal.

The remaining physical examination reveals no abnormal findings.

Radiology:

X-ray Hip a.p. and lateral left:

Pertrochanteric femur fracture on the left with avulsion of the greater trochanter. Preserved articulation of the hip joints. Vasosclerosis. Degenerative changes at the lumbosacral junction and ISG osteoarthritis.

X-ray wrist right a.p. and lateral:

Distal, intra-articular (< 2mm step-off), multi-fragmentary radius fracture tilted dorsally by approx. 20 degrees, post-traumatic ulna plus position, avulsion of ulnar styloid process

X-ray wrist right a.p. and lateral (after reposition):

Reduced intraarticular distal radius fracture with avulsion of the PSU in cast immobilization. Persistent, yet diminished angulation of the radiocarpal articular surface dorsally as well as shortening of the fracture compared to the previous examination. Improved Ulna-plus configuration.

Lab Results:

Complete Blood Count:

Test Name	Result	Normal Range
Hemoglobin	10.4	12.0 - 16.0 g/dL
White Blood Cell Count	10,800	4,500 - 11,000/μL
Red Blood Cell Count	4.0	4.2 - 5.4 million cells/μL
Platelet Count	213,000	150,000 - 450,000 cells/μL

Hematocrit	41.2	37.0 - 47.0%
Mean Corpuscular Volume (MCV)	90.5	80.0 - 100.0 fL
Mean Corpuscular Hemoglobin (MCH)	29.8	27.0 - 33.0 pg
Mean Corpuscular Concentration	32.9	32.0 - 36.0 g/dL

Metabolic Panel:

Test Name	Result	Normal Range
C reactive protein (CRP)	17.2	0.5mg/dl
Sodium (Na)	138	135 - 145 mmol/L
Potassium (K)	4.1	3.5 - 5.0 mmol/L
Calcium (Ca)	9.2	8.5 - 10.5 mg/dL
Chloride (Cl)	100	98 - 108 mmol/L
Bicarbonate (HCO3)	24	22 - 28 mmol/L
Blood Urea Nitrogen (BUN)	19	7 - 20 mg/dL
Creatinine	0.7	0.6 - 1.2 mg/dL
Glucose		70 - 99 mg/dL
Total Protein		6.4 - 8.3 g/dL
Albumin		3.5 - 5.0 g/dL

Coagulation:

Test Name	Result	Normal Range
Quick	110%	
International Normalized Ratio (INR)	0.9	0,8 – 1,2 INR

Prothrombin Time (PT)	12.1	10 - 14 seconds
Activated Partial Thromboplastin Time (aPTT)	24	25 - 35 seconds
Fibrinogen Level	300	200-400 mg/dL

Interdisciplinary Consultation to Hand and Wrist department:

Patient Name: Madame Joe

Age: 77

Consulting Department: Emergency Department

Consult: 77 yr patient with right intraarticular, distal radius fracture. Right-handed. Relatively reduced daily activity requirements. Please Advise regarding further imaging, surgical vrs conservative procedure.

Assessment:

Focused History:

Distal radius fracture after fall at home with contralateral concomitant pertrochanteric fracture. Patient describes low functional demand.

Physical Exam: evident bayonette deformity of the wrist, swelling and ecchymosis, as well as tenderness and reduced range of motion (flexion/extension, ulnar und radial abduction). No signs of nerve entrapment distally. Normal vascular status of the hand.

Imaging:

after reposition and cast immobilization, radial shortening >3mm, dorsal tilt from around 13°. Intra-articular displacement >2mm.

Plan:

Conservative treatment with cast inmobilisation for 6 weeks. Cast adjustments as needed. Begin with weight free ergotherapy after ruling out secondary dislocation in follow up X-ray (a.p. and lateral) in 1 week. Follow up consultation after 6 weeks in the outpatient clinic of hand surgery.

Admission plan:

- Admission to Trauma and Ortho
- Osteosynthesis prox. Femur left
- conservative treatment distal radius fracture right with cast 6 weeks

Progress notes

02.01.2023

Doctor’s Visit, Orthopedics and Traumatology

s: patient awake and alert, pain well managed, no shortness of breath, no chest pain, bedrest

o: afebrile vital signs stable, neurovascular intact hand and leg

a: pat doing well, surgery planed according to emergency program

p: conservative Tx wrist, Osteosynthesis with TFNA left for pertrochanteric femur fracture left today

Hospital day #2

Doctor’s Visit, Orthopedics and Traumatology

1 post operative day after TFNA left for pertrochanteric femur fracture left.

s: patient awake and alert, pain well managed, no shortness of breath, no chest pain, mobilization with physio only

o: wound dressing is dry, subcutaneous hematoma, ROM hip limited by pain, Fingers moving freely in cast, neurovascular hand and leg intact, afebrile, tachycardic, otherwise vital signs stable

a: pat doing well, surgery went according to plan, anemia w tachycardia, fist mobilization today

p: post op x ray, full weight bearing with physiotherapy and ergotherapy, transfusion 1 unit of blood

Complete Blood Count:

Test Name	Result	Normal Range
Hemoglobin	7.4	12.0 - 16.0 g/dL
White Blood Cell Count	11,800	4,500 - 11,000/µL
Red Blood Cell Count	3.5	4.2 - 5.4 million cells/µL
Platelet Count	238,000	150,000 - 450,000 cells/µL
Hematocrit	34.2	37.0 - 47.0%
Mean Corpuscular Volume (MCV)	90.8	80.0 - 100.0 fL
Mean Corpuscular Hemoglobin (MCH)	28.8	27.0 - 33.0 pg
Mean Corpuscular Concentration	33.9	32.0 - 36.0 g/dL

Metabolic Panel:

Test Name	Result	Normal Range
C reactive protein (CRP)	68.2	0.5mg/dl
Sodium (Na)	125	135 - 145 mmol/L
Potassium (K)	3.0	3.5 - 5.0 mmol/L
Calcium (Ca)	8.8	8.5 - 10.5 mg/dL
Chloride (Cl)	101	98 - 108 mmol/L
Creatinine	0.6	0.6 - 1.2 mg/dL
Blood Urea Nitrogen (BUN)	22	7 - 20 mg/dL

X-ray Hip a.p. and lateral left:

Status after treatment of a pertrochanteric femur fracture on the left with intramedullary nail. Intact material intact. No signs of loosening. No secondary dislocation. No new fracture. Preserved articulation. Subcutanoeus edema corresponding to surgical approach.

03.01.2023

2 post operative day after TFNA left for pertrochanteric femur fracture left

s: patient awake and alert, pain well managed, no shortness of breath, no chest pain, mobilization as tolerated

o: wound dressing is dry, subcutaneous hematoma w/o progression, ROM hip limited by pain, Fingers moving freely in cast, neurovascular, hand and leg intact, afebrile, vital signs stable

a: pat doing well, stable after transfusion, x ray ok, mobilization according to schedule

p: analgesia as needed, discharge to home, full weight bearing as tolerated, thromboprophylaxis with low molecular weight heparin (i.e. Clexane) until pain free mobilization, stitch removal after 2 wks, cast for 6 weeks and 5Kg weight bearing limit on the wrist, post op control with x ray in 6 weeks

Complete Blood Count:

Test Name	Result	Normal Range
Hemoglobin	9.1	12.0 - 16.0 g/dL
White Blood Cell Count	14,300	4,500 - 11,000/µL
Red Blood Cell Count	4.5	4.2 - 5.4 million cells/µL
Platelet Count	280,000	150,000 - 450,000 cells/µL
Hematocrit	37.2	37.0 - 47.0%
Mean Corpuscular Volume (MCV)	90.8	80.0 - 100.0 fL
Mean Corpuscular Hemoglobin (MCH)	28.8	27.0 - 33.0 pg
Mean Corpuscular Concentration	33.9	32.0 - 36.0 g/dL

2. Case 2: Middle aged man with acute traumatic Rotator Cuff tear

Hospital for Orthopedic Surgery

Patient Name: Doe, John

Gender: male

Age: 58 yrs

Ambulant Consultation Note from 12.02.2023

Diagnoses:

1. Full thickness Supraspinatus Tear Shoulder right (dominant)
   - Suspicion of labrum injury (anteroinferior)
   - After Bicycle Trauma one 05.02.2023

History of present illness**:**

A 58-year-old right-handed man reports pain in his right shoulder and collarbone after falling off his bicycle last week with outstretched hands. The pain is mainly in the right shoulder, is aggravated by any movement and subsides with Ibuprofen ice and rest. Nevertheless, after almost a week he hasn't been able to return to his work as an IT Technician due to pain. He denies numbness, tingling or weakness in his right arm. Additionally, he occasionally enjoys mountain biking and playing basketball and tennis.

Findings:

Physical Exam:

Shoulder right: Symmetrical shoulder relief, skin intact, no haematoma or swelling. Discrete diffuse tenderness on the glenohumeral joint, anteriorly and the intertubercular sulcus. Limited ROM due to pain with: anteversion/retroversion 90°/0°/20°, abduction/adduction 40°/0°/30°, external/internal rotation 15°/0°/90°. Starter’s, Jobe’s and O’Brien Test positive. Lift-off test negative, palm-up test negative, neer test negative. No piano key phenomenon, body cross test negative. Peripheral circulation, sensibility and motor function intact.

Radiographs Shoulder a.p., lateral and Neer:
Regular articulation with no evidence of dislocation, no evidence of fracture, no humeral head elevation or depression. Regular articulation of the AC joint.

Arthro-MRI of the right shoulder: practically full-thickness Tear of the supraspinatus tendon without muscle retraction or fatty infiltration. Additionally, suspicion a SLAP lesion with continuation of the rupture into the anterior labrum in the sense of a non-displaced Perthes lesion, no rupture of the rotator cuff, no HAGL lesion, no evidence of free joint bodies.

-

<u>Assessment and Plan:</u>
The above-mentioned findings in correlation with the history of trauma suggest an acute traumatic rupture of the supraspinatus tendon and possibly an accompanying Labrum injury. The patient can no longer actively raise the arm above the horizontal and is accordingly unable to work. In view of the overall situation with incapacity to work and significant pain, we recommend arthroscopic reconstruction of the rotator cuff rupture. In the same session a detailed inspection of Labrum integrity wll be

performed. If a rupture were to be found, a surgical fixation would be recommended. The patient agreed to the procedure. The operation is already planned for 20.02.2023 at our hospital.

Operation Note from 20.02.2023

Arthroscopic Rotator Cuff Repair Shoulder right

PROCEDURE: Primary right shoulder (dominant) arthroscopic rotator cuff repair (supraspinatus tendon) with subacromial decompression

INDICATION: This is a 58-year-old male. Refer to note in patient chart for documentation of history and physical. Due to the nature of the patient's increasing pain, surgery is recommended. The alternatives, risks and benefits of surgery were discussed with the patient. The patient verbalized understanding of the risks as well as the alternatives to surgery. The patient wished to proceed with operative intervention. A signed and witnessed informed consent was placed on the chart.

PRE-OP DIAGNOSIS:

1- Acute complete full thickness tear of the supraspinatus tendon right

2- Suspected anteroinferior labrum tear (Perthes)

POST-OP DIAGNOSIS: Acute complete tear of the supraspinatus, Shoulder impingement syndrome.

ANESTHESIA: General - Endotracheal.

FINDINGS:

LIGAMENTS / CAPSULE: Joint capsule within normal limits.

LABRUM: The labrum is within normal limits.

ROTATOR CUFF: Full thickness tear of the supraspinatus tendon, 5 mm anterior to posterior, by 10 mm medial to lateral. Muscles and Tendons: The biceps tendon is within normal limits.

JOINT: Normal appearance of the glenoid and humeral surfaces.

DESCRIPTION OF PROCEDURE:

PATIENT POSITIONING: Following induction of anesthesia, the patient was placed in the beach-chair position on the standard operating table with adequate padding on all prominent body parts. Subsequently, the surgical area was prepped and draped in the appropriate sterile fashion with Betadine.

INCISION TYPE:

1. Scope Ports: Anterior Portal.
2. Scope Ports: Posterior Portal.
3. Scope Ports: Accessory Anterior Portal.

INSTRUMENTS AND METHODS:

1. The arthroscope and instruments were introduced into the shoulder joint through the arthroscopic portals.

2. The subacromial space and bursa, biceps tendon, coracoacromial and glenohumeral ligaments, biceps tendon, rotator cuff, supraspinatus, subscapularis, infraspinatus, teres minor, capsulo-labral complex, capsule, glenoid labrum, humeral head, and glenoid, including the inner and outer surfaces of the rotator cuff, were visualized and probed.
3. The subacromial bursa, subacromial soft tissues and frayed rotator cuff tissue were resected and debrided using a motorized resector and 4.5 Synovial Resector.
4. The anterior portion of the acromion and acromial spur were resected with the 5.5 acromionizer burr. Approximately 5 mm of bone was removed. The coracoacromial ligament was released with the bony resection. The shoulder joint was thoroughly irrigated.
5. The edges of the cuff tissue were prepared, prior to the fixation, using the motorized resector.
6. The supraspinatus tendon was reattached and sutured using the arthroscopic knot pusher and the knotless anchor system and curved pointed suture passer and large bore cannula (to pass the sutures). The repair was accomplished in a side-to-side and a tendon-to-bone fashion using three double loaded suture anchors with 1 PDS suture.
7. The repair was stable to palpation with the probe and watertight.
8. The arthroscope and instruments were removed from the shoulder.
9. The skin was closed with 4-0 Vicryl using interrupted subcuticular technique.

DRAINS / DRESSING: Applied sterile dressing including gauze, iodoform gauze and Elastoplast.
SPONGE / INSTRUMENT / NEEDLE COUNTS: Final counts were correct.

INTRAOPERATIVE MEDICATIONS: No transfusions; minimal blood loss.

CAST / IMMOBILIZATION: The extremity was immobilized in a shoulder immobilizer.

POST-OP PLAN:

- Analgesic therapy as needed.
- Suture Removal 2 weeks postoperatively if wound healing is assured.
- Immobilization in abduction sling for 6 weeks postoperatively during the day and at night.
- Passive shoulder mobilisation starting at pod 1 with abduction/flexion up to 90°, external rotation up to 40°, internal rotation up to the abdomen for 6 weeks. No forced flexion and supination in the elbow for 6 weeks.
- Clinical follow-up 6 weeks postoperatively in our shoulder clinic.

Progress notes

-

21.02.2023

Doctor’s Visit, Orthopedics and Traumatology

1 pod after RC repair (supraspinatus tendon) with subacromial decompression right Shoulder

s: patient awake and alert, pain well managed with interscalene catheter, no shortness of breath, no chest pain, reports blurry vision

o: afebrile, vital signs stable, discreet ptosis of the left eye, rest of cranial nerves intact, arm immobilized in abduction sling, neurovascular of the arm intact (N. axillaris and musculocutaneus), wounds with no signs of inflammation and dry

a: pat doing well, surgery went well, suspicion of horner's syndrome

p: stop interscalene block ASAP, increased resp monitoring, weight free Mobilisation up to 90° with physio, discharge evt. Tomorrow if symptoms improve

22.02.2023

Doctor's Visit, Orthopedics and Traumatology

2 pod after RC repair (supraspinatus tendon) with subacromial decompression right Shoulder

s: patient awake and alert, pain well managed oral analgesia, no shortness of breath, no chest pain, vision is now normal

o: afebrile, vital signs stable, diminished ptosis of the left eye, rest of cranial nerves intact, arm immobilized in abduction sling, neurovascular of the arm intact (N. axillaris and musculocutaneus), wound dressing dry, mobilization went well

a: pat doing well, horner's symptoms sufficiently resolved

p: discharge today, postop indications as per op note

3. **Case 3: Middle aged man with an acute Achilles tendon rupture**

Local County Hospital

Emergency Chart

Patient Name: John Doe

Age: 45 yr

Gender: Male

Date of Admission: 01.01.2023

Diagnosis:

1. Achilles Tendon Rupture, right ankle

History of Present Illness:

The patient presented to the emergency department today complaining of a sudden onset of pain in the right calf while playing tennis some hours before. Afterwards, ambulation was impossible due to pain in the lower calf region. Since then, the whole ankle is clearly swollen. It is the first event of this nature. He is otherwise healthy, takes no medication, does leisure sport 1-3 week, smokes occasionally (socially), no known allergies.

Past Medical History:

Nonrelevant

Physical Exam:

Vital signs: Blood pressure 126/87 mmHg, heart rate 87 beats per minute, respiratory rate 16 breaths per minute, temperature 37°C/98.6°F.

Height: 176cm, Weight: 84.2 Kg; BMI 27.18

General appearance: Alert and oriented, in moderate distress due to pain.

Musculoskeletal examination: swelling and tenderness in distal calf region. A soft depression can be palpated along the Achilles tendon. No bony other bony tenderness, including the ankle. The Range of Motion is limited on both dosalextension and platarflexion. The Thomson test is pathologic (no plantarflexion while compressing the triceps surae). Neurovascular examination Intact.

Radiology Reports

X-ray of the ankle in ap (mortise) and lateral views:

Alignment is normal. No fracture. Soft tissue swelling over the lateral malleolus.

Emergency ultrasound:

Loss of continuity of the Achilles tendon ca. 3. cm away from insertion, a gap of ca. 1,5cm is visible.

Assesment/Plan:

The patient sustained an acute rupture of the right Achilles tendon. The MSK-Ultrasound suggests a rupture 3cm from the Calcaneal insertion. An ankle fracture was ruled out in x-ray. No lab abnormalities. After discussing the treatment

modalities, possible complications, post-surgical treatment and the expeted time to recovery, the patient decided to undergo surgical repair. The patient will be admitted and surgery will be performed tomorrow.

Entry Labs:

Complete Blood Count (CBC):

Test Name	Result	Normal Range
Hemoglobin	13.4	12.0 - 16.0 g/dL
White Blood Cell Count	7,800	4,500 - 11,000/μL
Red Blood Cell Count	4.5	4.2 - 5.4 million cells/μL
Platelet Count	200,000	150,000 - 450,000 cells/μL
Hematocrit	41.2	37.0 - 47.0%
Mean Corpuscular Volume (MCV)	90.5	80.0 - 100.0 fL
Mean Corpuscular Hemoglobin (MCH)	29.8	27.0 - 33.0 pg
Mean Corpuscular Concentration	32.9	32.0 - 36.0 g/dL

Metabolic Panel (CMP):

Test Name	Result	Normal Range
C reactive protein (CRP)	17.2	0.5mg/dl
Sodium (Na)	141	135 - 145 mmol/L
Potassium (K)	4.1	3.5 - 5.0 mmol/L
Calcium (Ca)	9.2	8.5 - 10.5 mg/dL
Chloride (Cl)	100	98 - 108 mmol/L

Bicarbonate (HCO3)	24	22 - 28 mmol/L
Blood Urea Nitrogen (BUN)	16	7 - 20 mg/dL
Creatinine	0.9	0.6 - 1.2 mg/dL
Glucose		70 - 99 mg/dL
Total Protein		6.4 - 8.3 g/dL
Albumin		3.5 - 5.0 g/dL

Coagulation:

Test Name	Result	Normal Range
Quick	110%	
International Normalized Ratio (INR)	0.9	0,8 – 1,2 INR
Prothrombin Time (PT)	12.1	10 - 14 seconds
Activated Partial Thromboplastin Time (aPTT)	24	25 - 35 seconds
Fibrinogen Level	300	200-400 mg/dL

Operative Note from 02.01.23

Diagnosis:

Right Achilles tendon rupture

- 2cm dehiscence (ED Sonography of 13.02.2023).

Surgery: Achilles tendon Repair, right ankle

Surgeons: Dr. X and Z

Anaesthesia:Regional anaesthesia, isolated

Anaesthetist: Dr. A

Indication:

The patient suffered a traumatic Achilles tendon rupture with >2 cm dehiscence seen on the emergency sonography. A shared decision for surgical treatment was taken together with the patiet. Informed consent was signed, a printed copy is available.

Technical procedure:

Patient in prone position. Sterile washing and draping. Team timeout. Cefuroxime singleshot. Incision medial to the Achilles tendon, dissection down to the peritendineum, which is opened longitudinally. Preparation to the Achilles tendon, which shows a dehiscence of >2 cm in the rupture zone.

Debridement is performed with subsequent slinging proximally and distally using the Krackow technique, followed by suturing and transfer suturing with PDS 1-0. Fine adaptation with Monocryl 3-0. Suture of the peritendineum (Monocryl 3-0). Irrigation and subcutaneous suture with Monocryl 3-0, skin suture with Prolene 4-0 in single button technique. Sterile dressing, and dorsal plaster splint.

Operating time:1 hr 6 min.

Postoperative procedure:

-Mobilisation for 2 weeks in lower leg plaster in pointed foot position.

-Follow-up after 2 weeks with removal of sutures and transition to Künzli shoe.

-Partial weight bearing with 15 kg for the next 6 weeks

-Physiotherapy starting after suture removal with passive mobilisation of the OSG

-Thrombosis prophylaxis with Fragmin fix for 6 weeks postoperatively

-Follow-up (clinical) in the orthopaedic consultation in 6 weeks

Progress Notes:

03.01.23

Doctor’s Visit, Orthopedics and Traumatology

1 post operative day after Achilles Tendon repair

s: patient awake and alert, pain well managed, no shortness of breath, no chest pain

o: ankle fixed with dorsal plaster splint, wound without inflammation signs and dry

a: pat doing well, surgery went according to plan, fist mobilization today

p: foot cast in equinus position, mobilization with partial weight bearing 15Kg with physiotherapy, discharge tomorrow is possible

B. Cases Sweden

1. Case 1: Woman with a pertrochanteric hip fracture

Emergency department admission note, 2023-06-16, 10.00, Dr Z

Social security number 19250101-1122

Reason for admittance Suspected hip fracture.

Earlier/current diseases Unspecified dementia, hypertension, stroke with sequelae.

Social background Lives in a special care facility. Independently mobile.

Current event Woman who likely fell on her right side last evening on her way to the bathroom. Found on the floor this morning. Patient is non-verbal habitually.

Status

General condition Seems to complain of pain.

Physical condition Right hip: The leg is shortened and externally rotated.

Heart Normal rhythm and heart sounds.

Lungs Normal breathing sounds bilaterally.

Findings

EKG Sinus rhythm.

Radiology X-rays of right hip, AP and lateral projections: Pertrochanteric, comminuted fracture. Trochanter major dislocated approximately 1 cm laterally.

Lab results

Leukocytes (3,5-8,8)	10,2 x10^9/L
Hemoglobin (117-153)	118 g/L
Trombocytes (165-387)	170 x10^9/L
Creatinine (<90)	125 mikromol/L
Sodium (137-145)	133 mmol/L
Potassium (3,5-4,6)	4,8 mmol/L
APT-time (20-30)	26 s
PK (<1,3)	1,5 INR
CRP (<5)	75 (mg/L)
Myoglobin (25-58)	400 microg/L

Plan

Pertrochanteric femur fracture. Admitted for osteosynthesis. The right leg marked using a permanent marker. Perioperative medications prescribed including pain killers, antibiotics and thrombosis prophylaxis. Furthermore, 2000 ml Ringer-Acetate because of the increased myoglobin and acute renal failure.

Diagnosis S72.10, W1999

<u>Operation note 2023-06-17, 12.00, Dr X</u>

Main diagnosis	S72.10, W19.99
Procedure	NFJ59, NFJ19
Date	2023-06-17
Operator 1	Dr X
Operator 2	Dr Y
Preoperative assessment	Woman with unspecified dementia and stroke with sequalae living in a special care facility. Ambulatory. X-rays show a rather comminuted pertrochanteric hip fracture on the right side. Planned for osteosynthesis.
Description of procedure	Spinal. Periooperative X-ray. Three doses of Cloxacillin.<br><br>Reposition in traction table through traction and adduction. X-rays show acceptable conditions. Sterile dressings are applied.<br><br>The incision site is marked. Sharp through skin and fascia. Rod is applied at the top of trochanter major. One step reamer. The medullary nail is applied, 180 mm 125 degrees. Stab incision for the collum screw. Drill. A 100 mm screw is applied and locked proximally. Stab incision for the distal locking screw. A 32 mm screw is applied. X-rays saved. Vicryl in fascia and subcutaneously. Skin sutured using Ethilon.
Post operative plan	Free mobilization.

Removal of sutures in three weeks.

No return visits planned.

Progress note 2023-06-18 09.00, Dr A

POD 1 PTFF right side. X-rays show adequate conditions. NEWS 0. Post operative labs show hemoglobin of 95 which is accepted. Creatinine 120. She is prescribed 1000 ml of Ringer Acetate. Dressings are unaffected. Patient seems at peace. Plan for today is mobilization and removal of urinary catheter.

Progress note 2023-06-18 15.00, Dr A

Patient seems to complain of discomfort around her urethra. Urine test is positive for leukocytes. Patient is diagnosed with a urinary tract infection. She is prescribed Pivmecillinam for 5 days. Not yet adequately mobilized, referral to geriatric department tomorrow?

Progress note 2023-06-19 12.00, Dr A

Lab results today show that creatinine is normalized. Still acceptable hemoglobin. Patient has been assessed twice by the physiotherapist. She has limited rehabilitation potential. The ward nurse will contact her special care facility and arrange for transportation home this afternoon.

2. **Case 2: Man with quadriceps rupture**

<u>Emergency department admission note, 2023-02-02, 10.00, dr A</u>

Social security number	19500101-2211
Reason for admittance	Rupture of quadriceps tendon.
Earlier/current diseases	Diabetes type 2, insulin treated. BMI 35.
Social background	Lives in an apartment. Self-sufficient. Ambulatory.
Current event	Yesterday evening the patient was outside his home shoveling snow. He slipped on a patch of ice and hyperextended his right knee. He crawled back inside and made it to bed. This morning he noticed swollenness around his knee and an ambulance was contacted. He has not been able to walk since the accident.

Status

General condition	Unaffected.
Physical condition	Right knee: generally swollen and tender. Superiorly to the patella one can palpate a diastasis in the quadriceps tendon. Patient is unable to flex his hip while extending his knee.
Heart	Normal rhythm and heart sounds.
Lungs	Normal breathing sounds bilaterally.

Findings

EKG Sinus rhythm.

Radiology X-rays of right knee, AP and lateral projections: No fracture. Light degenerative changes on the distal, lateral femur.

Lab results

Leukocytes (3,5-8,8)	9 x10^9/L
Hemoglobin (117-153)	135 g/L
Trombocytes (165-387)	210 x10^9/L
Creatinine (<90)	80 mikromol/L
Sodium (137-145)	143 mmol/L
Potassium (3,5-4,6)	4,8 mmol/L
APT-time (20-30)	26 s
PK (<1,3)	1,0 INR
CRP (<5)	30 mg/L

Plan

73-year-old diabetic with Quadriceps tendon rupture. Patient admitted to undergo tendon suture. F24. A knee orthosis is applied to keep the knee extended. The right leg is marked using a permanent marker. Perioperative medications prescribed including pain killers, antibiotics and thrombosis prophylaxis.

Diagnosis S76.1, W1999

Progress note 2023-02-03, 09.00, Dr B

Quadriceps tendon rupture awaiting tendon suture. NEWS 0. Patient declines pain while at rest. Unfortunately, the operation program is full today. F24.

Operation note 2023-02-04, 11.00, Dr C

Main diagnosis	S76.1, W1999
Procedure	NFL49
Date	2023-02-08
Operator 1	Dr C
Preoperative assessment	Man who fell while shoveling snow outside his home. Clinically diagnosed with a quadriceps tendon rupture in the emergency department. Planned for reinsertion of the tendon using suture anchors.
Description of procedure	General anesthesia. 2 g of Cloxacillin. Incision ventrally along patella and about 10 cm proximal of the knee joint. The rupture of the tendon is identified approximately 2 cm proximal of the patella. The respective ends of the tendon are cleared of debris using a scalpel. Three suture anchors are applied. Two Krackow-sutures are utilized in the proximal end of the tendon. They are thereafter tied to the anchors. The tendon is properly adapted. Wound closure using Vicryl subcutaneously and Ethilon in the skin.

Post operative plan	Orthosis 10 weeks. Initially locked to keep the knee extended for 2 weeks, thereafter unlocked step by step every two weeks. Free mobilization in the orthosis. Removal of the sutures in the orthopedic outpatient clinic in 2.5 weeks. Follow up visit in 10 weeks.

Progress note 2023-02-05, 10.30, Dr B

POD 1 suture for quadriceps tendon rupture. NEWS 0. Patient complains of pain in his right leg. Inspection of the knee shows unaffected dressings. However, the calf has an increased circumference compared to the other leg. He is also tender along the veins posteriorly in the calf. An ultrasound is ordered.

Progress note 2023-02-06, 11.00, Dr B

Ultrasound shows a DVT distal to the knee. The coagulation consultant recommends Apixaban for at least 3 months. The patient should be referred to the coagulation outpatient clinic when he is discharged.

The patient is mobilized successfully. He should be able to go back home tomorrow.

Case 3: Patient with septic arthritis

Emergency department admission note, 2023-01-01, 08.00, Dr A

Social security number	19600101-2233
Reason for admittance	Suspected septic arthritis.
Earlier/current diseases	Hypothyroidism. Elective THA left side 2015. No hips problems reported. Native knee joints.
Social background	Professional hunter.
Current event	2 days ago the patient was in the forest outside town hunting for game. He slipped on a rock and bumped his right tibia on a tree. He was able to complete his hunt. When he awoke the next day, his knee was swollen and he had had suspected fever chills during the night. This morning he was unable to make it out of bed on his own and an ambulance was contacted.

Status

General condition	Chills. Temperature around 39 degrees Celsius.
Physical condition	Right knee: Swollen, red, tender. Unable to flex.
	Right lower leg: wound measuring around 1-2 cm approximately 15 cm proximal to the ancle. No signs of infection.
Heart	Normal rhythm and heart sounds.

Lungs Normal breathing sounds bilaterally.

Findings

EKG Sinus rhythm.

Radiology X-rays of right knee, AP and lateral projections: No fracture. No degenerative changes.

Lab results

Leukocytes (3,5-8,8)	14 x10^9/L
Hemoglobin (117-153)	130 g/L
Trombocytes (165-387)	217 x10^9/L
Creatinine (<90)	80 mikromol/L
Sodium (137-145)	143 mmol/L
Potassium (3,5-4,6)	4,2 mmol/L
APT-time (20-30)	22 s
PK (<1,3)	1,2 INR
CRP (<5)	250 mg/L

Plan

Suspected septic arthritis. Sterile arthrocentesis is performed. Approximately 40 ml of cloudy, yellowish fluid is aspirated. Lab analysis of the synovial fluid shows

leukocytes >70. The sample is sent for culture. Blood cultures are taken. Thereafter the patient receives 2 g of Cloxacillin. Patient is admitted for irrigation.

Diagnosis M00.0G

<u>Operation note 2023-01-01, 09.30, Dr X</u>

Main diagnosis	M00.0G
Procedure	NGS19
Date	2023-01-01
Operator 1	Dr X
Preoperative assessment	Professional hunter who suffered a minor wound to his lower leg 2 days ago. Since then, signs of septic arthritis in his right knee have developed.
Description of procedure	General anesthesia. 2 g of Cloxacillin. Sterile dressings. Stab incision for the camera port laterally and an outflow port medially. Around 100 ml of pus is removed through suction. The knee is flushed using 5000 ml of NaCl. Moderate general synovitis is noted.
Post operative plan	Repeat irrigation in 2 days. Lab tests including CRP and WBC tomorrow.

Progress note 2023-01-02, 10.00, Dr B

Septic arthritis that underwent irrigation yesterday. NEWS 1. CRP 200, WBC 11. His temperature is still increased - around 37,8 degrees Celsius. Patient is tired and has no appetite. He is prescribed 1000 ml of glucose. Planned for second irrigation tomorrow.

Operation note 2023-01-03, 11.00, Dr X

Main diagnosis	M00.0G
Procedure	NGS19
Date	2023-01-03
Operator 1	Dr X
Preoperative assessment	Patient with septic arthritis who underwent irrigation 2 days ago. Blood cultures from the ED have come back positive for Staphylococcus aureus sensitive to Cloxacillin. Planned for a second irrigation today.
Description of procedure	General anesthesia. 2 g of Cloxacillin. Sterile dressings. Stab incisions in the same places as last time - camera port laterally and an outflow port medially. There is no pus in the joint. The knee is flushed using 5000 ml of NaCl. Moderate general synovitis is noted.

Post operative plan	No further irrigation is currently planned. Lab tests including CRP and WBC tomorrow. Referral to the infectious diseases clinic?

<u>Progress note 2023-01-03, 12.00, Dr B</u>

Second irrigation yesterday. Patient is feeling well. NEWS 0. Labs are improving - CRP 100, WBC 8. No further irrigation is currently necessary.

The infectious diseases consultant is contacted. She recommends Cloxacillin intravenously for a total of 7 days followed by Flucloxacillin orally for at least 6 weeks. The patient is accepted for transfer to the infectious diseases clinic.

C. Key Performance Parameters

The evaluation criterias included:

- Medical Accuracy: the comprehensiveness of the information provided of each major section of the documents, namely Diagnosis, History of Present Illness, Hospital Course and Discharge Plan; ensuring that all relevant aspects of the case are adequately addressed.
- Language: text style and vocabulary fit the situation and the indented target group that will read the text
- Conciseness: the text contains only the strictly necessary information, and follows a logical or chronological order
- Presence of Hallucinations: any text that is factually incorrect or inconsistent, or unrelated to the case
- Validity for clinical use: the text is deemed as usable in a true clinical scenario dealing with real patient information
- Possibility of bias: An assessment of the presence of convincing medical misinformation or biases that could exacerbate health disparities

D. Evaluation Form

1. Name and Last Name
2. Experience Level
    1. Resident
    2. Junior Attending (< 5 yrs as certified orthopedic surgeon)
    3. Senior Attending (5 yrs and longer as certified orthopedic surgeon)
3. Select the version of the Case:
    1. A
    2. B
4. **Medical Accuracy (Diagnoses):** are all relevant diagnoses correctly listed?
    1. There are major factual errors or inaccuracies present (missing or wrong diagnosis, misinterpretation of diagnostic testing)
    2. The information is generally accurate, but with a few minor errors
    3. The information completely accurate, no errors detected
    4. Other (please specify)
5. **Medical Accuracy (History of present illness):** are all relevant aspects adequately addressed?
    1. There are major factual errors or inaccuracies present (missing or wrong diagnosis, misinterpretation of diagnostic testing)
    2. The information is generally accurate, but with a few minor errors
    3. The information completely accurate, no errors detected
    4. Other (please specify)
6. **Medical Accuracy (Hospital course):** are all relevant events and their treatment correctly listed?

   1. There are major factual errors or inaccuracies present (missing or wrong diagnosis, misinterpretation of diagnostic testing)
   2. The information is generally accurate, but with a few minor errors
   3. The information completely accurate, no errors detected

7. **Medical Accuracy (Plan after discharge):** are all relevant instructions regarding treatment correctly listed?
   1. There are major factual errors or inaccuracies present (missing or wrong diagnosis, misinterpretation of diagnostic testing)
   2. The information is generally accurate, but with a few minor errors
   3. The information completely accurate, no errors detected
   4. Other (please specify)
8. **Language:** Is the language and style of the text clear enough to be easily understood by its intended audience?
   1. The text is not understandable for desired target group, several corrections are needed
   2. The text is generally understandable for target group, only minor corrections are needed
   3. The text is understandable for target group, no corrections are needed
   4. Other (please specify)
9. **Conciseness:** Does the text include only the essential information and present it in a logical or time-sequential manner?
   1. Text is poorly summarized (too long or too short), does not follow a chronological or logical order
   2. Text is acceptably summarized (could be more extended/concise), generally follows a chronological or logical order

3. Text accurately summarized, text is acceptably summarized (could be more extended/concise), generally follows a chronological or logical order
4. Other (please specify)

10. **Approval for clinical use:** is the text is deemed as usable in a true clinical scenario dealing with real patient information?
    1. Not approved for clinical use
    2. Needs correction, but could be used for clinical use
    3. Approved for clinical use
    4. Other (please specify)
11. Are there any factually incorrect or inconsistent or nonsensical text (known as hallucinations) ?
    1. Yes
    2. No
12. How would you rate the overall quality of the document, 0 being the lowest quality and 10 being the highest possible quality?

E. Prompt

1. "Chat, you are an orthopaedic and trauma surgeon. You will be provided with a text that represents the electronic medical health record of an orthopaedic patient. You then need to write a Discharge note based on this electronic medical health record. Say yes when you have understood this."

2. Insert the patient case as you have received it with all the information.

3. "That is a great first draft. I will now provide you with a description of the format of the discharge note used in our institution. The brackets indicate that information must be filled in. Importantly, these notes are meant to inform the family doctor of the hospital stay, therefore, a medical language should be used. If you have understood, proceed to adapt the previous patient's discharge letter to this format (Swedish format):

Discharge Summary

Care Period: (Date of Admission – Date of Discharge),

Orthopaedic Clinic Surgery Date:

Surgeons (List of all the surgeons)

Main Diagnosis: (main diagnosis relevant to the treatment)

Secondary Diagnosis Operation:

Past medical history:

Hospital course:

Discharge Instructions:

Discharge Medications (Medication List, including past medications and newly prescribed medications. Include dose and administration route following this format: Name, Xmg, oral, x times a day)"

4."Great. Now write a discharge letter for the patient in patient-friendly language. Use the following format: Discharge Letter (Date)

Subject: Discharge Letter of (Patient’s Name and Date of Birth)

Dear (Patient's Name), We are writing to you regarding your hospital stay in our institution from (date of admission) until (date of discharge).

Clinical Summary (short narrative in about 250 words or less of key information of the patient’s hospital stay, including investigation results, diagnoses, management and follow up instructions)"

F. Example manually generated notes

1. Discharge note

Treatment time: 230616-230619

Reason for enrollment: suspected right hip fracture

Operation date: 230617

Operator: doctor x, doctor y

Diagnosis: pertrochanteric fracture right

Procedure: Osteosynthesis with intramedullary marrow nail

History: 98-year-old lady with unspecified dementia, hypertension, previous stroke with sequelae, who fell in the bathroom of her residence during the night and was found on the morning of June 16, 2023. The patient could not be mobilized and presented to the emergency department. X-ray examination showed pertrochanteric fracture high side. Pain relief and thromboprophylaxis were given. She was scheduled to get surgery with a short medullary nail.

Course of care: Postoperative X-ray was adequate. The patient remained hemodynamically stable during the period of care. Because her creatinine was 120, she received 1000 mL ringer acetate. The wound dressing was dry. On day 2 postop, the patient developed a urinary tract infection and pivmecillinam was started for 5 days. Difficulties with mobilization led to referral to geriatrics. The physiotherapist assessed that the patient had low rehabilitation potential, thus she was discharged to her accommodation on the 3rd day postoperatively, her creatinine had normalised.

Planning: full load and free mobilization right side allowed. Suture removal in 3 weeks via VC. Pivmecillinam for 4 days from the date of discharge.

---

2. Discharge letter

Dear patient's name

You have been treated with us at the local emergency department in [hospital name] because you had broken your right hip. Your hip was operated on June 17th with a metal nail to hold the fracture together. Now the hip is repaired and you it bear your full body weight; note that it will hurt for the first few weeks.

We have given you an intravenous drip because you have been dehydrated. After the operation, you developed a urinary tract infection and we have prescribed penicillin for you, which you must take every day for 4 more days.

In 3 weeks, you must make an appointment with your health center so that they can help you remove the sutures.

Sincerely

doctor's name

G. Example ChatGPT 4 generated notes

1.Discharge Summary

Care Period: June 16, 2023 – June 19, 2023

Orthopedic Clinic Surgery Date: June 17, 2023

Surgeons: Dr. X, Dr. Y

Main Diagnosis: Pertrochanteric, comminuted fracture of the right hip (S72.10, W19.99)

Secondary Diagnosis Operation:

Acute Renal Failure

Urinary Tract Infection (UTI)

Past Medical History:

Unspecified dementia

Hypertension

Stroke with sequelae

Hospital Course:

The patient was admitted with a suspected right hip fracture and underwent osteosynthesis on June 17, 2023. Antibiotics, thrombosis prophylaxis, and analgesics administered. Post-operative labs showed acceptable levels of

hemoglobin and normalized creatinine. Urinary tract infection diagnosed and treated. Attempted mobilization post-surgery but with limited success.

Discharge Instructions:

Continue antibiotics (Pivmecillinam) for UTI as prescribed.

Mobilization as tolerated; however, the patient has been assessed to have limited rehabilitation potential. Sutures to be removed in 3 weeks. No return visits to this department planned. Referral to the geriatric department for further evaluation is advised.

Discharge Medications:

Cloxacillin, 500mg, IV, Perioperative (completed)

Pivmecillinam, 200mg, oral, 3 times a day for 5 days (for UTI)

Pain management as per the special care facility's protocol

Ongoing medications for hypertension and dementia (specific medications and doses to be continued as prior to hospital admission)

2.Discharge Letter

Date: June 19, 2023

Subject: Discharge Letter of Patient’s Name and Date of Birth

Dear Patient's Name,

We are writing to you regarding your hospital stay in our institution from June 16, 2023, until June 19, 2023.

Clinical Summary:

You were admitted to our hospital due to a suspected hip fracture after a fall at home. We confirmed the fracture through X-rays and you had surgery on June 17 to repair it. Your surgery went smoothly, and we used antibiotics and other medicines to make sure you're comfortable and safe from infection. After the surgery, you also had a urinary tract infection, which we're treating with antibiotics.

Your kidney function was a bit off initially, but it has since improved. The levels of your other tests, including your blood counts, have been closely monitored and are within an acceptable range. We tried to get you up and moving but noticed that you're not quite ready for intense rehabilitation. Therefore, we're sending you back to your special care facility, where you can continue to recover.

Follow-Up Instructions:

Please continue to take the antibiotics for your urinary tract infection as prescribed.

Your sutures (stitches) will need to be removed in three weeks. This can be done at your local healthcare facility.

We have no plans for you to return to our department, but a referral to the geriatric department for further evaluation may be beneficial for you.

Thank you for entrusting us with your care, and we wish you a speedy recovery. If you have any questions or concerns, please don’t hesitate to reach out to your healthcare team.

Best regards,

Your Doctor

Provider Signature:
